# Recent Advances in Ophthalmic Interventions: Exploring Innovations in Myopia, Cataract Surgery, and Visual Function

**DOI:** 10.3390/jcm14010195

**Published:** 2025-01-01

**Authors:** Kazuno Negishi

**Affiliations:** Department of Ophthalmology, Keio University School of Medicine, Tokyo 160-8582, Japan; kazunonegishi@keio.jp

As our understanding of ophthalmic optics and visual function advances, the intersection between clinical practice and technological innovation becomes increasingly significant in enhancing patient outcomes. This Special Issue of the *Journal of Clinical Medicine* highlights the latest research in this dynamic field. The six articles featured contribute valuable insights that can inform clinical decision making and patient care strategies within ophthalmology.

The global rise in myopia and other refractive errors has emerged as a pressing public health concern [1,2]. Factors such as increased screen time and reduced outdoor activities, particularly in East Asia, are exacerbating this trend [3,4,5]. In response, there has been a surge in innovative technologies and interventions aimed at preventing myopia progression [6]. In this issue, *Torii et al.* (Contribution 1) present a randomized pilot clinical trial investigating the effects of violet light (VL) emitted from eyewear devices on myopic children. Their findings suggest that VL irradiation may reduce axial elongation and choroidal thinning in children aged 8 to 10 years, indicating its potential as a non-invasive optical intervention in managing childhood myopia.

The evolution of cataract surgery techniques and intraocular lenses (IOLs) has significantly improved surgical safety and visual outcomes, with precise refractive correction now playing a pivotal role in achieving optimal results. Previous studies have shown that segmented axial length (AL) measurements enhance the accuracy of IOL calculations, particularly in eyes with long axial lengths [7], and, more recently, in both eyes with long and with short axial lengths [8]. *Goto et al.* (Contribution 2) assessed the effectiveness of segmented AL measurements in improving IOL power calculation accuracy for eyes with extremely long axial lengths. Their results demonstrated that segmented AL measurements enhanced the prediction accuracy of several new-generation IOL formulas, especially in high myopia cases, confirming previous findings. However, it is important to note that segmented AL measurements reduced the prediction accuracy of the Kane formula. Additionally, the study’s limitations, such as including both eyes from a single patient in some cases, should be considered. Despite these limitations, the findings offer valuable guidance for ophthalmic surgeons aiming to optimize refractive outcomes during cataract surgery.

Regarding IOLs, *Noguchi et al.* (Contribution 3) compared the visual performance of violet-filtered and blue-filtered intraocular lenses. The study suggests that violet-filtered IOLs may provide better contrast sensitivity under varying light conditions, although differences in optical platforms between the compared IOLs present a limitation. Furthermore, *Noguchi et al.* (Contribution 4) explored the relationship between anterior chamber depth (ACD) and refractive outcomes in patients who underwent scleral intraocular lens fixation (ISF). Their findings indicate that deeper IOL fixation correlates with reduced refractive error and a lower risk of iris capture. While these results serve as useful references for surgeons when selecting IOLs, they should be interpreted cautiously, as the retrospective nature of the study and potential influences from group classifications, patient backgrounds, and limited sample sizes may impact the findings.

As vitreoretinal surgery becomes increasingly less invasive, retinal detachment procedures are evolving from vision-preserving operations to those that also prioritize postoperative visual quality. *Okamoto et al.* (Contribution 5) examined factors affecting stereopsis after retinal detachment surgery, identifying visual acuity and contrast sensitivity as key predictors for stereopsis recovery. Given that stereopsis impairment can significantly affect daily activities like depth perception while driving, the study underscores the importance of comprehensive visual function assessments in postoperative care.

Beyond ophthalmic optics research, *Takigawa et al.* (Contribution 6) focused on the visual function in amblyopia, particularly the persistence of aniseikonia after treating anisometropic amblyopia. Through spatial aniseikonia testing, the authors revealed that aniseikonia persists even after successful amblyopia treatment, with no significant difference between amblyopic and anisometropic groups. These findings challenge previous assumptions about visual system adaptability and highlight the need for the ongoing management of aniseikonia in pediatric patients.

The contributions in this Special Issue reflect the dedication of researchers and clinicians striving to enhance the safety, efficacy, and accuracy of various ophthalmic interventions. I am confident that such research efforts will continue to drive the next wave of innovation in ophthalmology.
